# Alkaloid from *Geissospermum sericeum* Benth. & Hook.f. ex Miers (Apocynaceae) Induce Apoptosis by Caspase Pathway in Human Gastric Cancer Cells

**DOI:** 10.3390/ph16050765

**Published:** 2023-05-18

**Authors:** Mirian Letícia Carmo Bastos, João Victor Silva-Silva, Jorddy Neves Cruz, Amanda Roberta Palheta da Silva, Alexandre Augusto Bentaberry-Rosa, Gisele da Costa Ramos, José Edson de Sousa Siqueira, Márlia Regina Coelho-Ferreira, Sandro Percário, Patrícia Santana Barbosa Marinho, Andrey Moacir do Rosario Marinho, Marcelo de Oliveira Bahia, Maria Fâni Dolabela

**Affiliations:** 1 Post-Graduate Program in Biodiversity and Biotechnology, Federal University of Pará, Belém 66075-110, PA, Brazil; mirian.c.bastos@hotmail.com (M.L.C.B.); percario@ufpa.br (S.P.); 2 Post-Graduate Program in Pharmaceutical Sciences, Federal University of Pará, Belém 66075-110, PA, Brazil; jorddynevescruz@gmail.com; 3 Laboratory of Medicinal and Computational Chemistry, Institute of Physics of São Carlos, University of São Paulo, São Carlos 13563-120, SP, Brazil; jvssilva89@gmail.com; 4 Faculty of Pharmacy, Federal University of Pará, Belém 66075-110, PA, Brazil; amandaroberta247@gmail.com (A.R.P.d.S.); rosa.alexandref@gmail.com (A.A.B.-R.); 5 Post-Graduate Program in Chemistry, Federal University of Pará, Belém 66075-110, PA, Brazil; giamajesus@gmail.com (G.d.C.R.); siqueira.edson@outlook.com (J.E.d.S.S.); pat@ufpa.br (P.S.B.M.); andrey@ufpa.br (A.M.d.R.M.); 6 Emílio Goeldi Paraense Museum, Coordination of Botany, Ministry of Science, Technology, Innovation and Communications, Belém 66077-830, PA, Brazil; mcoelho@museu-goeldi.br; 7 Laboratory of Human Cytogenetic, Institute of Biological Sciences, Federal University of Pará, Belém 66075-110, PA, Brazil; mbahia@ufpa.br; 8 Post-Graduate Program in Pharmaceutical Innovation, Federal University of Pará, Belém 66075-110, PA, Brazil

**Keywords:** indole alkaloid, cancer, apoptosis, necrosis, caspase

## Abstract

Gastric cancer is among the major causes of death from neoplasia leading causes of death worldwide, with high incidence rates and problems related to its treatment. Here, we outline how *Geissospermum sericeum* exerts antitumor activity on the ACP02 cell line (human gastric adenocarcinoma) and the mechanism of cell death. The ethanol extract and fractions, neutral fraction and alkaloid fraction, were characterized by thin-layer chromatography and HPLC-DAD, yielding an alkaloid (geissoschizoline N4-methylchlorine) identified by NMR. The cytotoxicity activity of the samples (ethanol extract, neutral fraction, alkaloid fraction, and geissoschizoline N4-methylchlorine) in HepG2 and VERO cells was determined by MTT. The ACP02 cell line was used to assess the anticancer potential. Cell death was quantified with the fluorescent dyes Hoechst 33342, propidium iodide, and fluorescein diacetate. The geissoschizoline N4-methylchlorine was evaluated in silico against caspase 3 and 8. In the antitumor evaluation, there was observed a more significant inhibitory effect of the alkaloid fraction (IC50 18.29 µg/mL) and the geissoschizoline N4-methylchlorine (IC_50_ 12.06 µg/mL). However, geissoschizoline N4-methylchlorine showed lower cytotoxicity in the VERO (CC_50_ 476.0 µg/mL) and HepG2 (CC_50_ 503.5 µg/mL) cell lines, with high selectivity against ACP02 cells (SI 39.47 and 41.75, respectively). The alkaloid fraction showed more significant apoptosis and necrosis in 24 h and 48 h, with increased necrosis in higher concentrations and increased exposure time. For the alkaloid, apoptosis and necrosis were concentration- and time-dependent, with a lower necrosis rate. Molecular modeling studies demonstrated that geissoschizoline N4-methylchlorine could occupy the active site of caspases 3 and 8 energetically favorably. The results showed that fractionation contributed to the activity with pronounced selectivity for ACP02 cells, and geissoschizoline N4-methylchlor is a promising candidate for caspase inhibitors of apoptosis in gastric cancer. Thus, this study provides a scientific basis for the biological functions of *Geissospermum sericeum*, as well as demonstrates the potential of the geissoschizoline N4-methylchlorine in the treatment of gastric cancer.

## 1. Introduction

Cancer is one of the leading public health problems worldwide, responsible for about one in six deaths [1,2]. Cancer incidence rates increase with population aging and exposure to risk factors such as smoking, excessive alcohol consumption, lack of physical activity, and obesity [1]. Estimates point to approximately 29 million cancer cases worldwide in 2040, an increase by 47% compared to 2020. Countries in transition are the most affected due to demographic changes, increased associated risk factors to globalization, and a growing economy [3]. Once diagnosed, patients have a long way to go and may undergo surgical procedures, radiotherapy, and/or systemic therapy (chemotherapy, hormone treatments, and targeted biological therapies) [2].

Gastric cancer (adenocarcinoma) is the fourth leading cause of cancer death, constituting 5.6% of all new cases, with the fifth highest incidence among cancers worldwide, and with more than one million new cases diagnosed annually [1]. Treatment selection considers the stage of gastric cancer, with chemotherapy used in advanced tumor cases [4]. However, there are certain problems associated with chemotherapy treatment, such as tumor resistance to treatment [5], high toxicity [6], and high cost [7].

**Figure 1:**
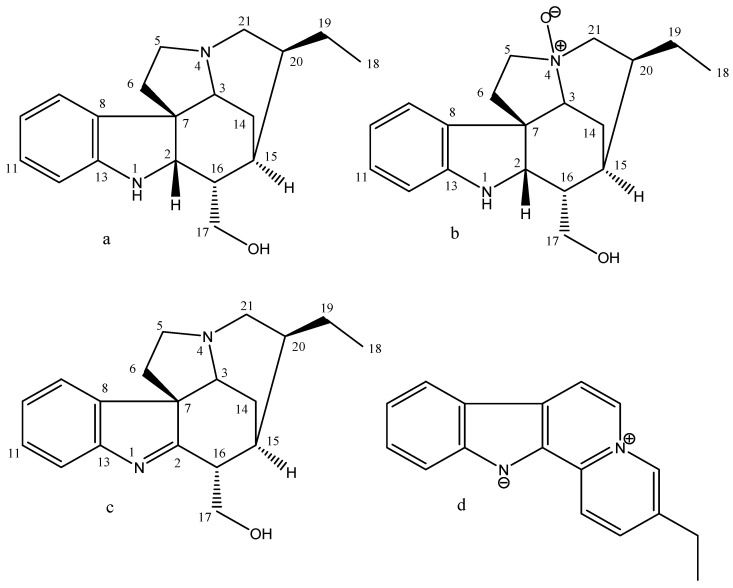
Alkaloids isolated from *Geissospermum sericeum*. (**a**) geissoschizoline, (**b**) geissoschizoline N4-oxide, (**c**) 1,2-dehydrogeissoschizoline, (**d**) flavopereirine.

In this context, plants can be a valuable contribution to the search for new therapeutic alternatives, especially plant species that produce alkaloids [8]. These molecules have a low molecular weight and account for approximately 20% of the secondary metabolites found in plants [9]. Traditionally, the bark decoction of *Geissospermum* has been used in the Amazon region to treat malaria, fever, and stomach disorders [10]. Extracts from the bark and leaves of *Geissospermum*, including ethanolic [11], methanolic [10], and hydroalcoholic [12], have been tested for biological activities, including antitumor activity [13]. One species that synthesized promising alkaloids for antitumor activity is *Geissospermum sericeum* Benth. & Hook.f. ex Miers (Apocynaceae). The MeOH−H_2_O (9:1) extract of the bark of *G. sericeum* yielded geissoschizoline, geissoschizoline N4-oxide, 1,2-dehydrogeissoschizoline, and flavopereirine alkaloids (Figure 1) [12].

The flavopereirine, dihydroflavopereirine, and PB-100 (mixture of flavopereirine and dihydroflavopereirine) were submitted to evaluate antitumor activity using different tumor and standard strains. The 100 µg/mL concentration of PB-100 inhibited 16 other tumor cell lines, including drug-resistant ones, and did not inhibit the multiplication of normal cells (maintained 95% of normal cells viable). Flavopereirine and dihydroflavopereirine showed similar antitumor activity, confirming the hypothesis that these two alkaloids are responsible for the activity of PB-100 [14]. Flavopereirin is a β-carboline alkaloid, and according to Beljanski and Beljanski [15], alkaloids belonging to the β-carboline class can inhibit DNA synthesis of cancer cells while leaving healthy DNA unaltered, potentially providing a cancer treatment with reduced side effects [13]. Different mechanisms may be involved in the antitumor activity of alkaloids, such as the interaction with microtubules [16]; induction of apoptosis [17]; induction of apoptosis by activating caspases, and altering the Bcl-2/Bax ratio [18].

Despite studies on isolating alkaloids from *Geissospermum sericeum*, none of the approaches addresses the isolation of geissoschizoline N4-methylchlorine and its activity in gastric cancer. The purpose of this study is to describe for the first time the isolation of geissoschizoline N4-methylchlorine in this species. It investigates whether apoptosis may be the mechanism involved in the activity of geissoschizoline N4-methylchloride. Furthermore, it evaluates the impact of the fractionation of the ethanolic extract of *G. sericeum* on antitumor activity and cytotoxicity.

## 2. Results

### 2.1. Phytochemical Analysis

The friction process made it possible to obtain a 2.2% yield of ethanol extract (EE) from the barks of *G. sericeum* (Table 1). This extract was subjected to fractionation by acid-base partition, obtaining a neutral fraction (NF, 17.3%) and a fraction of alkaloids (AF, 10.7%; Table 1).

**Figure 2:**
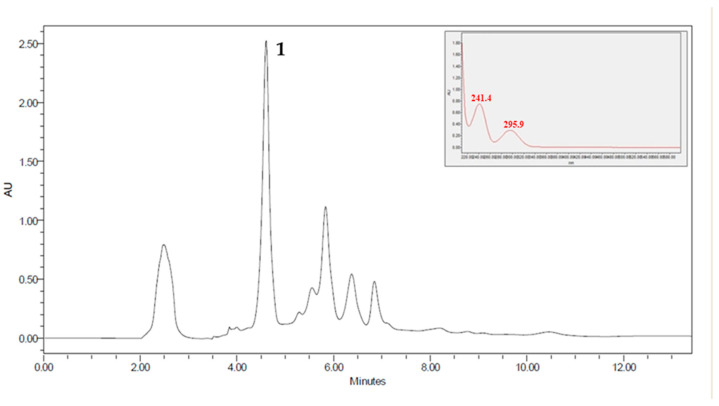
Chromatographic HPLC profile of the subfraction F6AF obtained from the stem barks of *G. sericeum*. (**1**) geissoschizoline N4-methylchlorine isolated compound. Condition: SunFire™ Prep. C18 OBD™ 5 µm 19 × 150 mm column, flow rate = 10.0 mL/min, temperature 18 °C. Mobile phase—t = 0 min: 80% water and 20% acetonitrile; t = 10 min: 80% water and 20% acetonitrile.

**Figure 3:**
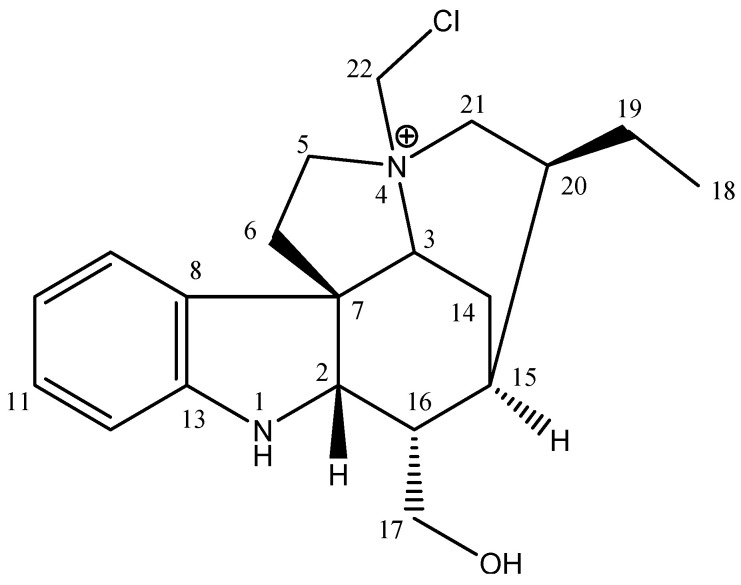
Compound structure of geissoschizoline N4-methylchlorine (**1**).

Studies in thin-layer chromatography suggest that EE, NF, and AF must contain alkaloids. Due to less complexity, the AF was subjected to fractionation on a Sephadex LH-20 column, giving rise to 18 subfractions. Semi-preparative HPLC analyzed subfraction 6 (F6AF), and the chromatogram obtained showed a peak around 4.5 min with absorption at λ = 241 and 295 nm, compatible with the indole system. Then the height was collected to isolate compound **1** (Figure 2 and Figure 3; Table 2).

Through electrospray ionization mass spectrometry (ESIMS) and nuclear magnetic resonance (NMR) data, it was determined that its molecular formula was C_20_H_28_N_2_OCl (Supplementary Materials Figures S1–S3). In the ^13^C NMR spectrum of compound **1**, 20 carbon signals, three signals from no hydrogenated carbon atoms, nine from methine, seven from methylene, and one from a methyl carbon atom were observed according to DEPT experiments. In the ^1^H NMR spectrum of compound **1,** signals typical of an indole alkaloid [12] at δ 7.29 (d, 7.5, ^1^H, H-9), δ 6.80 (t, 7.5, ^1^H, H-10), δ 7.08 (t, 7.5, ^1^H, H-11) and δ 6.65 (d, 7.7, ^1^H, H-12) were observed. The signal observed for H-2 at 4.03 (d, 5.5), together with the HMBC correlations observed from H-9 and H-2 to a quaternary carbon atom at δ 53.4 (C-7), indicated that compound **1** is a dihydroindole derivative with an extended fused ring system. Moreover, the presence of characteristic resonances was observed for C-17 CH_2_OH (δ 3.64 and 3.79; δC 65.6) and C-19–C-18 Et group (δ 1.02 (CH_3_) and 1.33 (CH_2_a) and 1.46 (CH_2_b); δC 11.5 and 23.6, respectively). The CH_2_OH group was attached at C-16 by the HMBC correlations of H-17a and H-17b with carbons C-2, C-16, and C-15 signals. The Et group was localized at C-20 by the HMBC correlations of H-19 with C-15, C-18, C-20, and C-21 signals. The NMR data suggest that compound **1** is structurally similar to the indole alkaloid geissoschizoline, with some chemical shift changes to carbons C-3, C-5, and C-21 [12].

However, the MS spectrum showed 49 additional mass units for compound **1**, compatible with the CH_2_Cl group. Through the mass pattern characteristic for chlorine, its presence was confirmed in compound **1**, *m/z* 347 [MCl_35_] (100) and *m/z* 349 [MCl_37_] (30). The pattern of HMBC correlations observed for **1** was identical to geissoschizoline, with changes observed due to the additional CH_2_Cl group. The CH_2_Cl group was localized at N-4 through HMBC correlation of CH_2_Cl (δ 5.59 and 5.40) with the carbons C-5 (δ 64.2), C-21 (δ 54.5), and C-3 (δ 77.0). NMR data to compound **1** are shown in Table 2. Thus, the compound **1** was identified as geissoschizoline N4-methylchlorine (Figure 3).

### 2.2. Antitumor Activity, Cytotoxicity, and Selectivity Index

Gastric cancer cells (ACP02) were submitted to treatment with ethanol extract (EE); neutral fraction (NF); alkaloid fraction (AF), and the alkaloid geissoschizoline N4-methychlorine (compound **1**). It was witnessed that the fractionation of EE led to obtaining AF, where alkaloid **1** showed similar significant activities in this cell line (Table 3).

The standard renal cell line (VERO) was also treated in the same manner as the ACP02. In contrast to what was observed for the tumor cell, alkaloid **1** showed a lower cytotoxic effect for the VERO cells, and its selectivity was high for the tumor cell. It should be considered that the fractionation contributed significantly to the increase in the selectivity index. Furthermore, the EE, NF, AF, and alkaloid **1** cytotoxicity were evaluated in human hepatoma (HepG2), showing the lower toxicity of the alkaloid and higher selectivity for activity in gastric tumor cells (Table 3).

### 2.3. Assessment of Apoptosis Induction

AF and alkaloid 1 were more active in ACP02, so they were submitted to the assay to evaluate cell death by apoptosis or necrosis using doxorubicin as a positive control. Cell death by apoptosis was observed in both AF and alkaloid **1** treated cells.

**Figure 4:**
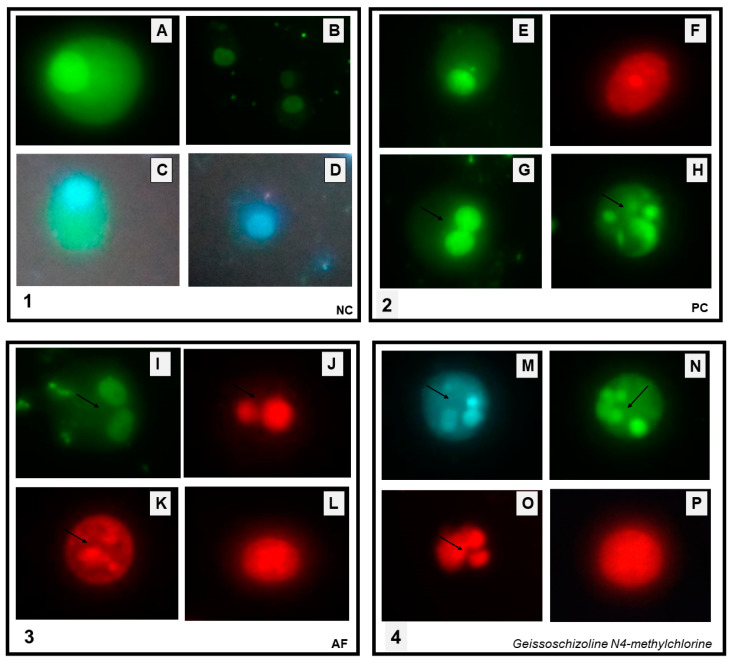
Micrographs of ACP02 cells after exposure to doxorubicin, AF, and geissoschizoline N4-methylchlorine for 48 h. 1—Negative control (NC): (**A**–**D**)—viable cells with intact nuclei and cytoplasm. Presence of cells stained with Hoechst 33342 (HO) that binds to DNA staining the nucleus in blue and with fluorescein diacetate (DAF) staining the cytoplasm in green. 2—Cells treated with the positive control (PC; doxorubicin, 1 µM) (**E**) viable cells, (**F**) necrotic cells stained red by propidium iodide (PI); (**G**,**H**) apoptotic cells with emphasis on the presence of apoptotic bodies (arrows). 3—Cells treated with the alkaloid fraction of *G. sericeum* (AF, 18 µg/mL): (**I**–**K**)—apoptotic cells with emphasis on the presence of apoptotic bodies (arrows); (**L**)—necrotic cells stained red by propidium iodide. 4—Cells treated with isolated indole alkaloid (geissoschizoline N4-methylchlorine, 12 µg/mL): (**M**–**O**)—apoptotic cells with the presence of apoptotic bodies (arrows), (**P**)—necrotic cells stained red by PI.

Apoptotic bodies were visualized in AF-treated cells (Figure 4I–K). Whereas some cells showed no change in the cell membrane, suggesting an early stage of apoptosis (Figure 4I), others already showed membrane changes, possibly in the final stage of apoptosis (Figure 4K). It was also possible to observe permeability changes in some of the cells (Figure 4, quadrant 3 J). In cells treated with alkaloid **1**, the following changes were observed: apoptotic bodies (Figure 4M–O); changes in the cell membrane and permeability (Figure 4O); no membrane alteration suggesting apoptosis in the early stages. Similar changes were seen in doxorubicin-treated ACP02 cells (Figure 4G,H).

**Figure 5:**
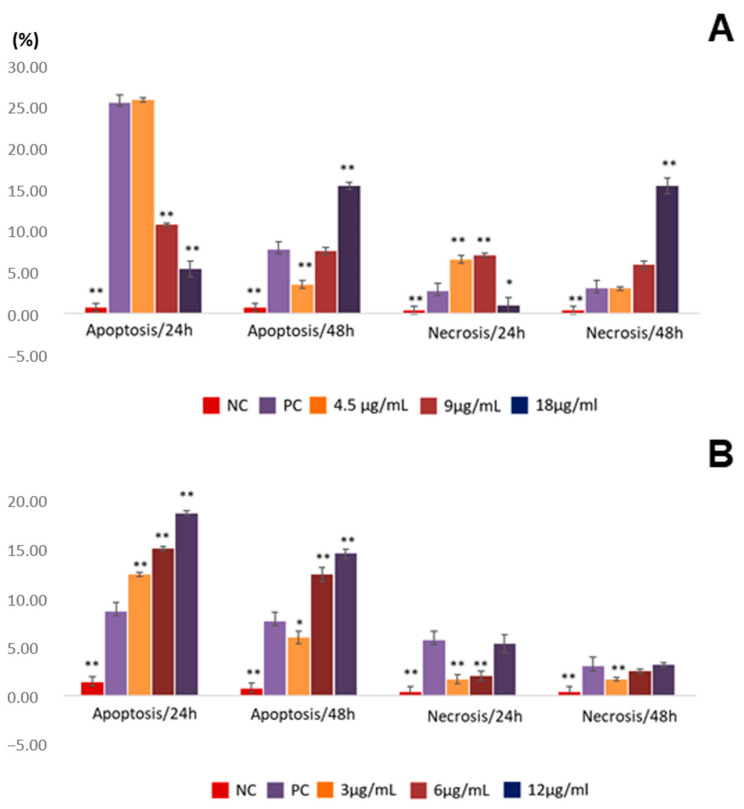
Frequency of apoptosis and necrosis in ACP02 cells exposed to AF and geissoschizoline N4-methylchlorine. (**A**) alkaloid fraction of G. *sericeum* (AF); (**B**) geissoschizoline N4-methylchlorine. NC, negative control; PC, positive control. * *p*< 0.05; ** *p* < 0.01. The data represent the mean ± standard deviation. * *p*  <  0.05 and ** *p*  <  0.001.

The lowest concentration of AF induced apoptosis in a proportion equal to that of doxorubicin, approximately 25%, with no statistically significant difference (Figure 5). With increasing concentration, apoptosis and necrosis decrease. With an increase in exposure time (48 h), there is a reduction in apoptosis and necrosis at the lowest concentration and an increase in the proportion of apoptosis and necrosis at the highest concentration (Figure 5).

Differently from the alkaloid fraction, the proportion of apoptosis induced by geissoschizoline N4-methylchlorine at times of 24 and 48 h was concentration-dependent, higher at 12 µg/mL. The highest percentage of apoptosis was observed after 24 h of exposure. The induction of cell death by necrosis was less pronounced at both analyzed times (Figure 5).

### 2.4. In Silico

The favorable binding mode of the geissoschizoline molecule N4-methylchlorine with the pocket binding of caspase-3 and 8 was chosen according to the Moldock score results. The Moldock score values are summarized in Table 4.

**Figure 6:**
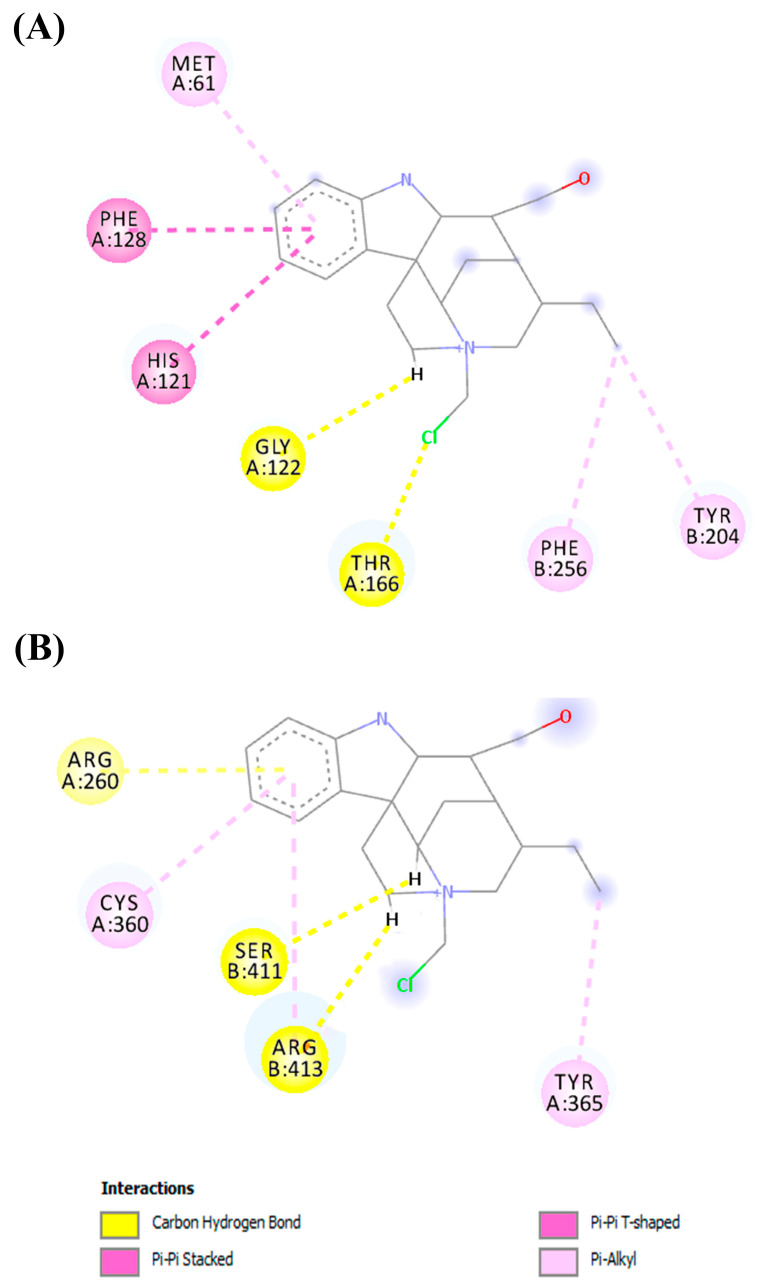
Interactions were established between geissoschizoline N4-methylchlorine and the binding pocket of (**A**) caspase-3 and (**B**) caspase 8.

Figure 6 shows the interactions in the complex between the geissoschizoline N4-methylchlorine and caspases 3 and 8. In the binding cavity of the two molecular targets, the ligand interacted with its residues. With caspase 3, the ligand interacted through hydrophobic interactions with the residues of Met61, Phe128, His121, Phe256, and Tyr204. The interactions were Pi-Pi stacked with Phe128, His121, and Pi-Pi T-shaped with Phe256 and Tyr204. In addition, hydrogen bonds were established with Gly122 and Thr166. In the complex formed between geissoschizoline N4-methylchlorine and caspase-8, hydrophobic interactions of the Pi-Alkyl type were formed with Cys360 and Tyr365. Another three hydrogen bonds were formed with Arg260, Ser411, and Arg413.

**Figure 7:**
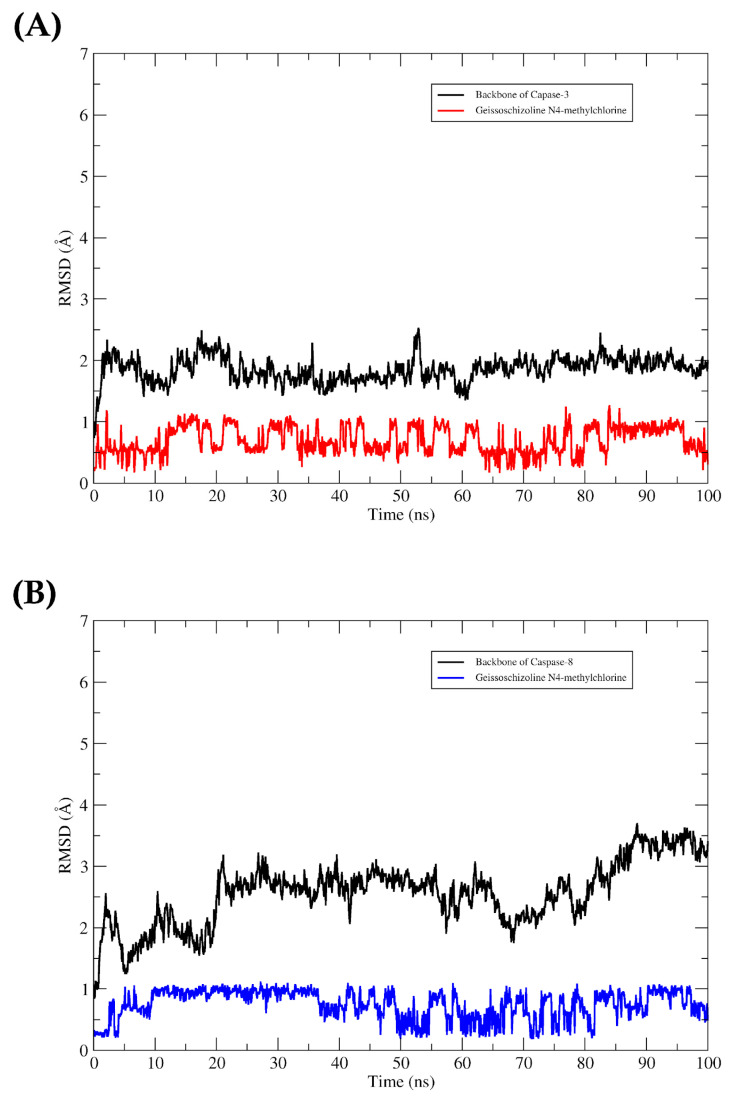
RMSD values for 100 ns of MD simulation. Complexes formed between geissoschizoline N4-methylchlorine and (**A**) caspase-3 and (**B**) caspase 8.

The RMSD plots showed conformational stabilities of caspases-3 and 8 to the geissoschizoline ligand N4-methylchlorine throughout the 100 ns trajectory, as the complexes remained formed. It was observed that throughout the entire trajectory, the complexes remained formed. The ligand in the two proteins’ binding cavity did not show large deviations in their plots. The time series of RMSD values are shown in Figure 7.

We used the MM-GBSA method to evaluate the system affinity energy after the MD simulations. It was observed that the van der Waals, electrostatic, and non-polar interactions were the ones that most contributed to the formation of the complex. Of these energies, the van der Waals interactions were the ones that most collaborated, reaching values of ΔE_vdW_ = −32.15 and ΔE_vdW_ = −30.64 kcal/mol for the caspase-3 and caspase-8 systems. The sum of all these energies also demonstrates that the complexes formed spontaneously, as observed in the values of ΔG_bind_ = −35.62 kcal/mol and ΔG_bind_ = −30.69 kcal/mol, for caspase-3 and 8, respectively. The values obtained are shown in Table 5.

## 3. Discussion

In the study of plants, whether the fractionation of the extract influences the biological and cytotoxic activity is considered. This study investigated the antitumor activity of EE, NF, AF, and alkaloid geissoschizoline N4-methylchlorine (compound **1**) samples against gastric cancer cells (ACP02), as well as the cytotoxic activity against VERO cells and HepG2 hepatoma cells.

The results showed that, in general, the VERO cell was more sensitive to the deleterious effect of *G. sericeum* than the HepG2 cells. On the other hand, the fractionation of the ethanol extract contributed to the increase in biological activity, especially of geissoschizoline N4-methylchlorin, with reduced cytotoxicity and increased selectivity index. This factor is significant because cancer chemotherapy often includes drugs that cause cytotoxic effects [19] causing adverse effects, and decreasing patient adherence to treatment, indicating the potential use of this compound in future clinical trials.

Literature data report other indole alkaloids such as geissoschizoline, geissoschizoline N4-oxide, and 1,2-dehydrogeissoschizoline, as well as a β-carboline alkaloid flavopereirine, obtained from the bark of *G. sericeum* showed activity for the human KB cell line. For the N4 oxide of geissoschizolina and geissoschizolin, the IC_50_ was >40 μM, while the IC_50_ of the 1,2-deiidrogeissoschizolina derivative was 40 μM. Flavoperericin also showed moderate cytotoxic activity (IC_50_ 10.7 μM); this alkaloid was the most active in this study. The cytotoxicity of flavopereirin has been cited previously due to its ability to selectively inhibit deoxyribonucleic acid (DNA) synthesis in tumor cells compared to healthy cells [12].

The alkaloid fraction obtained from the shells of *G. sericeum* made it possible to isolate compound **1**, with a high selectivity index for the gastric tumor cell. The results suggest that it is a promising compound for antitumor activity. This may occur because the fractionation of the extract impacts its activity and cytotoxicity [11]. When compound **1** is compared with the alkaloid geissoschizoline N4-oxide [12], it is observed that the oxide group was replaced by methylchlorine. Other studies have shown that halogenated substances contain a biological potential. These substances act as antibacterial, antifungal, antiviral, anti-inflammatory, antiproliferative, anti-fouling, anti-food, cytotoxic, ichthyotoxic, and insecticidal [20,21,22].

Studies of the antitumor activity of *Geissospermum* are still scarce. However, a report in the literature of an in vitro assay performed with *G. vellosii* (EGv) extract shows a dose- and time-dependent induction of apoptosis and selectively inhibiting ovarian cancer cell growth [13]. In another study, the evaluation of EGv in metastatic castration-resistant prostate cancer (CRPC) cells showed a concentration and time-dependent response of apoptosis induction and cell cycle arrest. The EGv-induced cell cycle inhibitors, p21 and p27, repressed PCNA, cyclin A, and cyclin D1. Furthermore, it induced upregulation of Bax (pro-apoptotic), reduction of Bcl-2 (anti-apoptotic), Bcl-xL, and XIAP expression, which are associated with cleavage of poly (ADP-ribose) polymerase [23]. Several indole alkaloids such as geissolosimine, geissospermine, geissoschizoline, geissoschizone, and vellosiminol have already been isolated with an elucidated structure from extracts of this species [10], which suggests a possible contribution to this activity.

Alkaloids used in cancer treatment, vinblastine and vincristine, can induce cell death by apoptosis in various tumor cells [24]. In the present study, AF and geissoschizoline N4-methylchlorine induced tumor cell death through apoptosis, and it has been observed that this occurs mainly within 24 h of exposure.

Naturally, the cell cycle is regulated by variable mechanisms because the cells are influenced by the tissue in which they are inserted. Gastric cells, for example, always renew themselves continuously in the cell cycle. However, this factor can be modified in cancer development [25]. This action may not be related only to the cell cycle of tumor cells since cancer cells’ molecular makeup and biological properties can vary significantly, even within the same tumor [26].

In our study, we used the exposure time factor to verify the influence of this aspect on the biological action and defense mechanisms of the cell. Based on its hypothesis, the lowest concentrations of the geissoschizoline N4-methylchlorine alkaloid activate the apoptosis pathway using tools of action similar to specific cyclo-cellular natural products, such as the vinca alkaloids.

The mechanisms involved in cell death by apoptosis are complex, and there are two interconnected signaling pathways: the extrinsic (or cell death receptor pathway) and the intrinsic pathway (or mitochondrial pathway), and molecules of one of them can influence the other [27]. One of the main protein sets responsible for apoptosis is caspases [28], and their activation leads to rapid cell death. Caspases that are involved in the process of apoptosis have been classified into two subtypes based on their mechanism of action: initiator caspases (caspase-2, -8, -9, and -10) and executioner caspases (caspase-3, -6, and -7) [29,30]. It has been reported that activation of caspase-8 and caspase-3 is involved in the mechanism of apoptosis induction in gastric cancer [31].

Several studies have reported the success of molecular docking methodology in investigating the interaction of compounds of natural origin with molecular targets of pharmacological interest (Costa et al., 2020; da Silva Júnior et al., 2021; Leão et al., 2020; Santos et al., 2020). In order to assess whether caspases activation is involved in the antitumor activity of geissoschizoline N4-methylchlorine, docking and molecular dynamics simulations were performed, using caspases 3 and 8 as enzymatic targets, as they are fundamental mediators of the intrinsic and extrinsic pathway of apoptosis, respectively [32].

Our molecular docking results showed that the compounds interact favorably with caspases 3 and 8. The investigation showed hydrophobic interactions between geissoschizoline N4-methylchlorine and caspase-3, with Met61, Phe128, His121, Phe256, and Tyr204. For caspase 3 the Met residue is part of a hydrophobic recess, formed together with Phe55 and Phe128, which harbors His121, and any change in these residues affects the enzyme’s activity [33]. Furthermore, this analysis revealed the selectivity of the alkaloid for caspases, as it interacted with hydrophobic residues in the S2 pocket (Tyr204, Trp206, and Phe256) that are unique to caspases 3 and 7 [34]. The interaction of geissoschizoline N4-methylchlorine with caspase 8 also formed hydrophobic interactions but with the residues of Cys360 and Tyr365. The catalytic triad residues Cys360, His317, and the carbonyl oxygen atom of Arg258 are present in the active site of caspase 8 [35,36]. Interactions with active site residues, separately or together, can generate consequent changes in the enzymatic mechanism [37]. In addition, hydrogen interactions also involved other residues in stabilizing the alkaloid and caspase-3 complexes further, as well as the alkaloid and caspase-8 complexes. In order to take into account the protein flexibility and evaluate the dynamic stability of the predicted interactions between the alkaloid and the caspases, the lowest energy conformations obtained from the coupling were submitted to MD simulations for 100 ns. The RMSD values showed that the complexes remained formed throughout the entire trajectory, and the scoring function analysis highlights the contribution of van der Waals interactions to the binding energy (alkaloid/caspases complex). These data implied the involvement of the intrinsic pathway accompanied by the activation of the extrinsic pathway in the induction of apoptosis during the treatment step with geissoschizoline N4-methylchlorine.

Previous studies have shown that alkaloids show binding affinity with the enzymes caspase-3 [38] and caspase-8 [39] in an in silico study, and that in vitro indole alkaloids induce apoptosis through activation of caspase-3 and 8 [40]. According to these reports, our results show that geissoschizoline N4-methylchlorine can interact reversibly with residues from the active site of caspases 3 and 8 and trigger an inhibitory response in gastric cancer cells, a hypothesis that corroborates the results obtained in vitro.

## 4. Materials and Methods

### 4.1. Reagents

Methanol-d4 was purchased from Cambridge Isotope Laboratories Inc. (Miami, FL, USA). Ethanol was purchased from Tedia (São Paulo, SP, Brazil). Roswell Park Memorial Institute 1640 medium (RPMI 1640), [3- (4,5-dimethylthiazol-2-yl) -2,5-diphenyltetrazolium] bromide solution (MTT), dimethylsulfoxide (DMSO), doxorubicin hydrochloride and fluorescein diacetate (FDA) were purchased from Sigma-Aldrich (St. Louis, MO, USA). Fetal bovine serum, phosphate buffered saline (PBS), and Trypsin were purchased from GIBCOTM (made in Brazil), while propidium iodide and Hoechst 33342 were purchased from Invitrogen™ (Carlsbad, CA, USA).

### 4.2. Equipment

CO_2_ incubator (Thermo Fisher Scientific, Waltham, MA, USA), microplate spectrophotometer reader (Thermo Fisher Scientific, Waltham, MA, USA), benchtop centrifuge (Benchtop Mikro 220R centrifuge, Hettich, Bäch, Switzerland), fluorescence microscope (Olympus BX41, Olympus Corporation, Tokyo, Japan). HPLC-DAD system (column SunFireTM C18 5 μm; 4.6 × 150 mm; Waters Co., Milford, MA, USA), LC-MS (ACQUITY TQD SYSTEM, Waters Corporation, Milford, MA, USA), and NMR Varian Mercury 300 (Varian, Oxford, UK), Sephadex LH-20 column (Sigma-Aldrich, St. Louis, MO, USA).

### 4.3. Plant Material, Extraction Procedure and Isolation

The stem barks from *Geissospermum sericeum* Benth. & Hook.f. ex Miers were collected at Vitória do Xingu region, State of Pará, Brazil (S 2°52′50.5″ W 52°00′29.1″) in 26 January 2013. The species was identified by technician L.C. Batista Lobato of the Emilio Goeldi Museum of Pará, and voucher specimen was incorporated in MG Herbarium (MG209580) of this institution.

The bark powder was macerated in an ethanolic solution, and the maceration product was concentrated in a rotary evaporator giving rise to the ethanolic extract. Then, the ethanolic extract was acid–base partitioned, obtaining the neutral fraction (FN) and the alkaloid fraction (FA). The AF underwent further fractionation in a chromatography column, where Sephadex LH-20 was used as the stationary phase, giving rise to 18 subfractions (F1AF-F18AF). The F6AF subfraction showed a chromatographic profile for alkaloids by TLC analysis. It was selected for alkaloid isolation by high-performance liquid chromatography in preparative mode on an HPLC-PAD Binary pump 1525 (Waters) equipped with PDA (2698 Waters), SunFire™ Prep. C18 OBD™ 5 µm 19 × 150 mm column, using flow rate = 10.0 mL/min, temperature 18 °C, mobile phase water/acetonitrile 8:2 for 12 min, resulting in the isolation of indolic alkaloid geissoschizoline N4-methylchlorine.

### 4.4. NMR and MS Analysis

The mass spectrum was obtained by direct infusion using negative ion mode on an electrospray ionization source (ESI). To record the NMR spectra, the sample was dissolved in methanol-d4. The chemical shifts are presented in delta (δ) values and the coupling constants (J) in Hertz (Hz).

### 4.5. Cells

For cytotoxic tests, used tumor lineage of primary gastric adenocarcinoma (ACP02), hepatocellular carcinoma (HepG2), and kidney cells of African green monkey kidney cells *Cercopithecus aethiops* (VERO) were used. These cells were supplied by the Human Cytogenetics Laboratory (LCH) of the Institute of Biological Sciences of the Federal University of Pará (UFPA). For the apoptosis assay, the tumor line ACP02 was used.

### 4.6. Antitumor Activity and Cytotoxicity through the Cell Viability Assay (MTT)

The cell viability assay was carried out according to the methodology described by Mosmann et al. [41]. Using 96-well plates, VERO cells were seeded (8 × 10^3^ cells/mL medium RPMI-1640); ACP02 (8 × 10^3^ cells/mL medium RPMI-1640), and HepG2 (1 × 10^4^ cells/mL medium RPMI-1640). The plates were incubated at 37 °C in a humid atmosphere with 5% CO_2_.

After incubation (24 h), the cells were treated in triplicate with seven concentrations (500–7.812 μg/mL) of the ethanol extract, neutral fraction, alkaloid fraction, and geissoschizoline N4-methylchlorine. A triplicate for negative control was also performed, containing only 10% fetal bovine serum. The plates were returned to the oven at 37 °C and 5% CO_2_ humidity for further incubation for 24 h. Then [3-(4,5-dimethylthiazol-2-yl) -2,5-diphenyltetrazolium bromide] (MTT, 5 mg/mL) solution was added to the wells containing the treatment samples. They were subsequently incubated at 37 °C at 5% CO_2_ for 4 h. The supernatant was discarded, and 100 µL of dimethylsulfoxide (DMSO) was added to all wells to dissolve formazan crystals. The plates were homogenized for the complete dissolution of the crystals. Around 1 h later, the absorbances of the wells were quantified in a microplate spectrophotometer reader working with a reference wavelength of 570 nm. Cell viability was expressed as a percentage of the control absorbance in the untreated cells after subtracting the appropriate background. Inhibitory concentration (IC_50_ for ACP02 cells) and cytotoxic concentration (CC_50_ for VERO and HepG2 cells) were determined by linear regression [11]. The selectivity index (SI) was obtained from the ratio between the IC_50_ values (Vero or HepG2) and the IC_50_ of the ACP02 cell.

### 4.7. Investigation of Necrosis and Apoptosis by Quantifying Cell Death Patterns

The cell lines from primary gastric adenocarcinoma (ACP02) (2.5 × 10^5^ cells) were incubated for 24 h in a 5% CO_2_ and 37 °C. After, the cells were treated with AF (4.5 μg/mL, 9 μg/mL and 18 μg/mL) and indole alkaloid (geissoschizoline N4-methylchlorine; 3 μg/mL, 6 μg/mL and 12 μg/mL), doxorubicin was used as possible control (1 μM and 1.84 µg/mL) and negative control only cells (2.5 × 10^5^) in culture medium. The cells were incubated again for 24 and 48 h in 5% CO_2_ at 37 °C. After that, the cells were washed with 3 mL phosphate-buffered saline (PBS; 2×), suspended in 3.0 mL of trypsin, and then centrifuged at 1500 rpm for 5 min. The supernatant was discarded, and the dye mix was prepared with a final volume of 100 μL (25 μL propidium iodide—PI; 50 μL fluorescein diacetate—FDA; 10 μL Hoechst 33342—HO and 15 μL PBS), whose concentrations were 5 μg/mL for PI, 15 μg/mL for FAD, and 2 μg/mL for HO. Then, 100 μL of the cell suspension was mixed into 2 μL of the dye mix, and the mixture was placed in a water bath at 37 °C for 5 min. Subsequently, in one slide, 15 μL of the cell suspension of each sample was added and covered with a coverslip. The cells were analyzed using an OLYMPUS BX41 fluorescence microscope containing three filters (DAPI/FITC/TRITC) by quantifying 300 cells per group. Cells were evaluated according to staining and morphology: in viable cells—green cytoplasm and intact blue nucleus; apoptotic cells—condensed and fragmented blue nucleus, condensed and fragmented red nucleus, without differentiation between early and late apoptosis; and necrotic cells—intact red nucleus. Viability cell determination was performed by dividing viable cells by total cells multiplied by 100.

### 4.8. Molecular Docking

Our studies used caspases 3 and 8 as target proteins for geissoschizoline N4-methylchlorine. The compound’s molecular structure was optimized with B3LYP/6-31G* [42] in Gaussian 09 [43].

To perform the docking simulation, we used the Molegro Virtual Docker software (MVD, version 5.5) [44] and the 3D structure used as the molecular target can be found in the PDB using ID: 2XYP (caspase 3) [45] and 3KJN (caspase 8) [46]. The MolDock Score (GRID) function was used with a grid resolution of 0.30 Å and a radius of 5 Å encompassing the entire connection cavity. A description of the method can be found in [47].

### 4.9. Molecular Dynamics (MD) Simulations

Ligand RESP loads were obtained with HF/6-31G [48], and parameters were created using Antechamber [49] and described by GAFF [50]. The MD simulations were performed using the Amber 18 [51,52,53,54]. The tLEaP module was used to add the missing hydrogens to the protein structures. The PDB2PQR server [55] was used to determine the protonation state of the residuals of caspases 3 and 8. The MD simulations were performed using the 14SB force field [56]. TIP3P water molecules [57] were used to solvate the systems in an octahedron periodic box. The distance for the shear radius was 12 Å for all directions. Counterions were added to neutralize the system charges.

Adapted energy minimization protocols, heating, and md production simulations can be found in [58]. The Particle Mesh Ewald method [59] was used to calculate the electrostatic interactions, and the bonds involving hydrogen atoms were restricted with the SHAKE algorithm [60]. The temperature control was performed with the Langevin thermostat [61] within a collision frequency of 2 ps^−1^.

### 4.10. Binding Affinities Calculations

For the estimation of the receptor–ligand affinity, energy was calculated with the molecular mechanics/generalized born surface area (MM/GBSA) method according to [62,63,64]. We used the calculations of the last 10 ns of the MD simulation.

The free energy was estimated according to Equation (1):

ΔG_bind_ = ΔE_MM_ + ΔGs_olv_ − TΔS (1)

### 4.11. Statistical Analysis

The results were submitted to the analysis of variance test (ANOVA), followed by the Tukey test of the Bioestat 5.0 program to compare the various parameters. In all analyses, the level of significance was *p* < 0.05. The cytotoxic concentration of 50% (CC_50_) and the inhibitory concentration of 50% (IC_50_) were calculated using the program GraphPad Prism version 6.0.

## 5. Conclusions

Bioguided investigation on the plant of *G. sericeum* showed that the ethanol extract was less active in inhibiting APC02 cells. However, fractionation improved the antitumor response, with the activity and selectivity observed for the alkaloid fraction. Notably, bioguided monitoring also resulted in the isolation of an indole alkaloid geissoschizoline N4-methylchlorine, for the first time described in the literature, with selective cytotoxicity in gastric tumor cells and involvement of apoptosis in the cell death process. Moreover, the study exploring possible mechanisms showed that the induction of cell death might be related to its interactions when coupled to caspase 3 and 8, presented in the docking results and maintained throughout the molecular dynamics. This observation suggests that the geissoschizoline N4-methylchlorine alkaloid may act as a caspase inhibitor, contributing to its antitumor activity.

However, additional validation studies utilizing in vivo models are necessary to substantiate the findings obtained in the present study. In addition, we highlight the scientific community’s interest in investigating natural products for developing new drugs, highlighting the role of the Amazon as a source of actives, especially from the Apocynaceae family, used in traditional medicine in the Amazon.

## Figures and Tables

**Table 1:** Phytochemical characterization of ethanol extract of *G. sericeum* and its fractions.

Samples	Yield (%)	TLC	
		UV Light	Dragendorff
EE	2.2	+	+
NF	17.3	+	+
AF	10.7	+	+

TLC, thin layer chromatographic; EE, ethanol extract; NF, neutral fraction; AF, alkaloid fraction.

**Table 2:** ^1^H- and ^13^C NMR data for F3F6FA (300 MHz, CD3OD).

Position	^1^H-NMR	^13^C-NMR	HMBC
1			
2	4.03 (d, 5.5)	64.3	C-3, C-7, C-15, C-16
3	3.72 (dd, 14.5, 4.2)	77.0	C-8, C-15, C-22
4			
5	3.85 (dd, 12.0, 6.8) 3.96 (ddd, 12.0, 11.8, 8.1)	64.2	C-3, C-7
6	2.66 (m)	35.1	C-7, C-8
7		53.4	
8		134.2	
9	7.29 (d, 7.5)	123.7	C-7, C-11, C-13
10	6.80 (t, 7.5)	121.0	C-8, C-12
11	7.08 (t, 7.5)	130.4	C-9, C-13
12	6.65 (d, 7.7)	111.4	C-8, C-10
13		151.0	
14	2.43 (dd, 15.0, 2.6) 1.87 (dt, 15.0, 3.1)	23.3	C-15
15	1.74 (br)	28.7	
16	2.16 (m)	34.8	C-17, C-20
17	3.64 (dd, 10.5, 6.8) 3.79 (dd, 10.5, 9,7)	65.6	C-2, C-15, C-16
18	1.02 (t, 7.4)	11.5	C-19, C-20
19	1.33 (m) 1.46 (m)	23.6	C-15, C-18, C-20, C-21
20	1.96 (m)	39.2	C-16
21	3.42 (t, 14.0) 3.71 (m)	54.2	C-20, C-22
22	5.40 (d, 10.0) 5.59 (d, 10.0)	67.1	C-3, C-5, C-21

The numbers in parentheses are J values in Hz.

**Table 3:** Antitumor activity (IC_50_), cytotoxicity (CC_50_), and selective index (SI) of *G. sericeum*.

Samples	ACP02	Vero Cells		HepG2 Cells	
	IC_50_ (µg/mL)	CC_50_ (µg/mL)	SI_ACP02_	CC_50_ (µg/mL)	SI_ACP02_
EE	131.55 ± 0.49	113.65 ± 0.35	0.86	472.55 ± 0.63	3.59
NF	114.45 ± 0.21	130.10 ± 0.42	1.13	259.30 ± 0.42	2.27
AF	18.29 ± 0.02	173.3 ± 0.32	9.48	299.45 ± 0.35	16.37
Geissoschizoline N4-methylchlorine	12.06 ± 0.04	476.0 ± 0.54	39.47	50.5 ± 0.28	41.75

Data represent mean ± SD of at least three experiments realized in triplicate. EE, ethanol extract; NF, neutral fraction; AF, alkaloid fraction; IC_50_: inhibitory concentration for 50% of cells; IC_50_: cytotoxic concentration for 50% of cells; SI = CC_50_ cells/IC_50_ cells.

**Table 4:** Moldock score results for the investigated complexes.

Compound	Molecular Target	
	Caspase-3	Caspase-8
Geissoschizoline N4-methylchlorine	−49.72	−56.31

**Table 5:** Binding energy values and energy components in kcal/mol.

Complex	ΔE_vdW_	ΔE_ele_	ΔG_GB_	ΔG_NP_	ΔG_bind_
Caspase-3	−32.15	−9.34	10.38	−4.51	−35.62
Caspase-8	−30.64	−8.84	12.42	−3.63	−30.69

ΔE_vdW_, contributions by van der Waals; ΔE_ele_, electrostatic energy in the gas phase; ΔGGB, polar solving energy; ΔG_np_, nonpolar solvation energy; ΔG_bind_, binding affinity.

## Data Availability

Data is contained within the article and supplementary material.

## Supplementary Materials

The following supporting information can be downloaded at: https://www.mdpi.com/article/10.3390/ph16050765/s1. Figure S1: ^1^H NMR spectrum of compound **1** (300 MHz, MeOD). Figure S2: ^13^C NMR spectrum of compound **1** (75 MHz, MeOD). Figure S3: Full scan MS spectrum of compound **1** ESI(+).
