# Supplementary material for: Alkaloid from Geissospermum sericeum Benth. & Hook.f. ex Miers (Apocynaceae) Induce Apoptosis by Caspase Pathway in Human Gastric Cancer Cells
Source: Pharmaceuticals (Basel). 2023 May 18;16(5):765. doi: 10.3390/ph16050765 (PMC10222982; doi:10.3390/ph16050765)

Figure S1. ^{1}H NMR spectrum of compound 1 (300 MHz, MeOD)

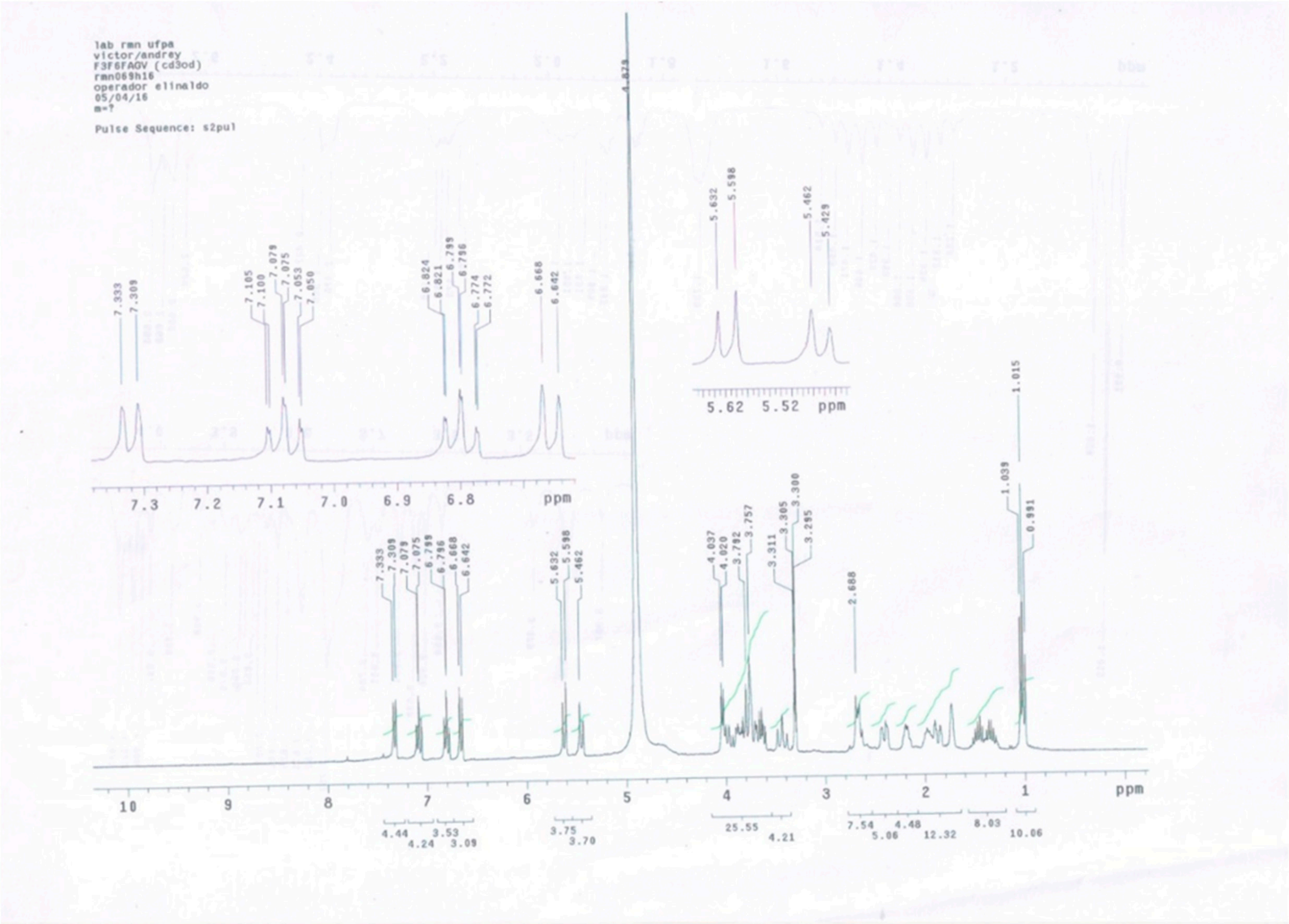

Figure S2. ^{13}C NMR spectrum of compound 1 (75 MHz, MeOD)

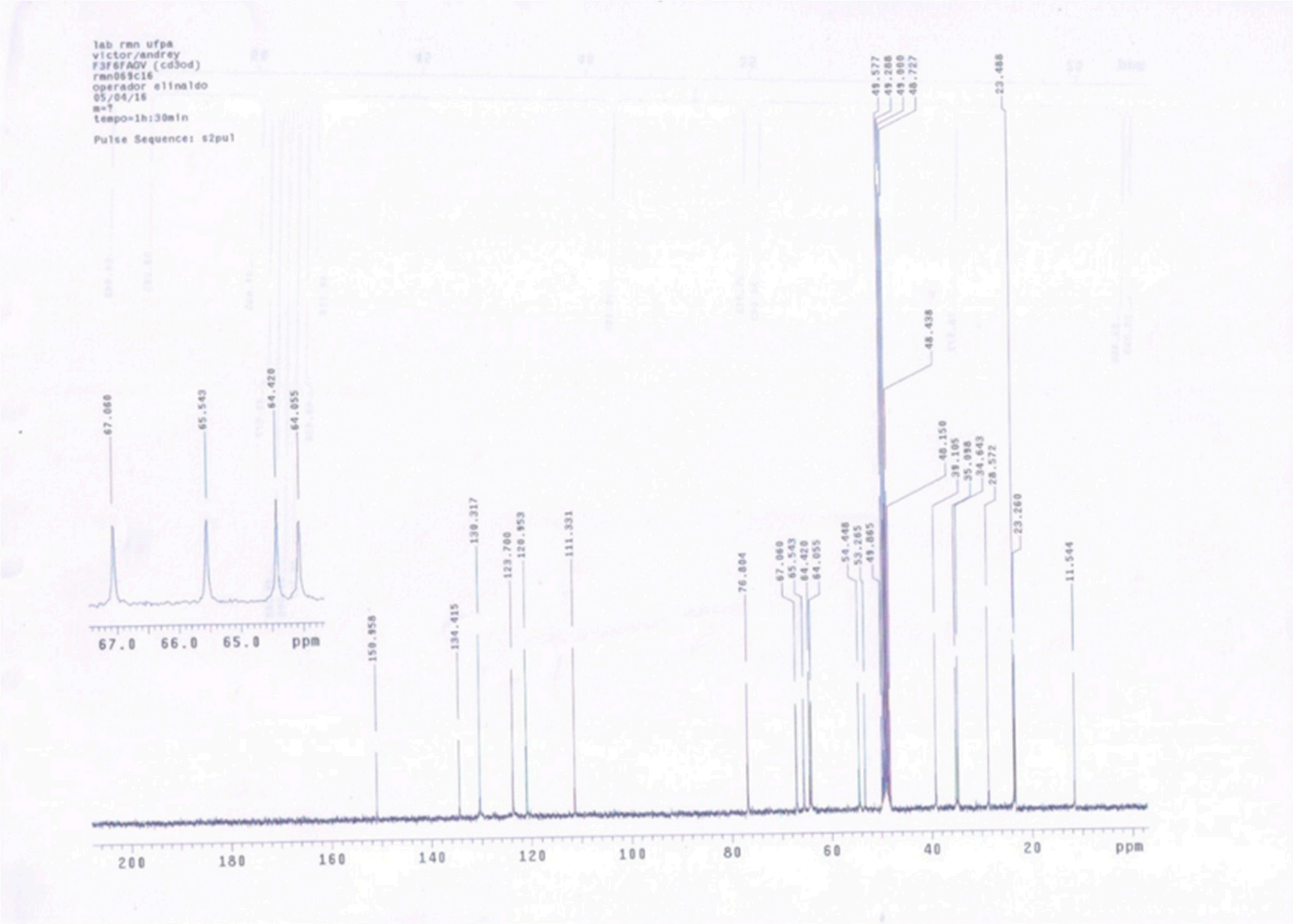

Figure S3. Full Scan MS spectrum of compound 1 ESI(+)

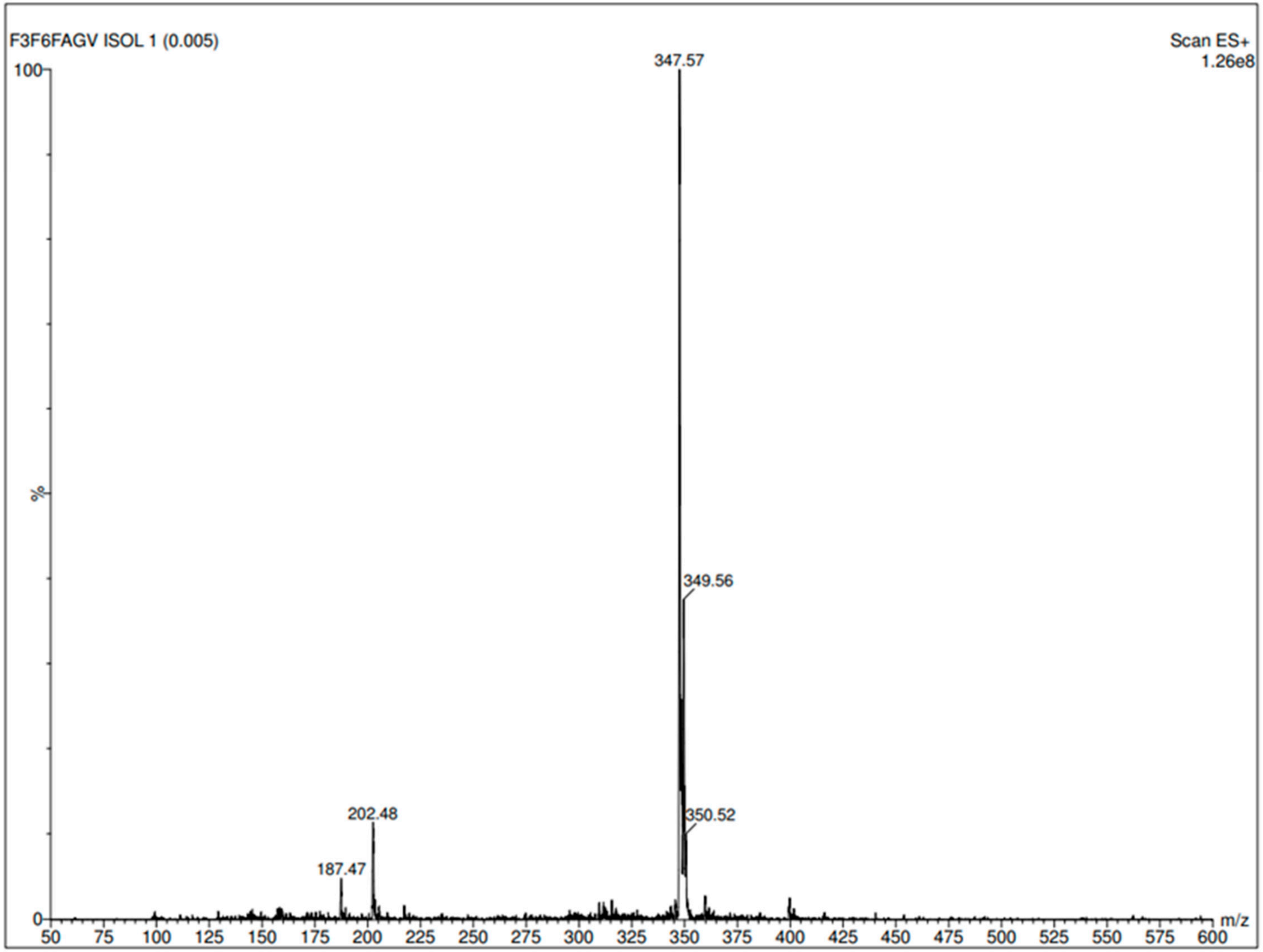

Supplement: Supplementary file 1 [file pharmaceuticals-16-00765-s001.zip › pharmaceuticals-2364675-supplementary.pdf]
